# Dynamic interdependence between consumer confidence and housing prices: Evidence from bootstrap rolling window causality tests

**DOI:** 10.1371/journal.pone.0340354

**Published:** 2026-01-12

**Authors:** Yumei Guan, Chiwei Su, Yunfeng Wang

**Affiliations:** 1 School of Management, University of Sanya, Sanya, Hainan, China; 2 School of Economics, Qingdao University, Qingdao, China; 3 Faculty of Finance, City University of Macau, Macao, China; University of the Witwatersrand Johannesburg, SOUTH AFRICA

## Abstract

This paper examines the bidirectional relationship between consumer confidence (CC) and housing prices (HP) using a bootstrap sub-sample rolling causality test. The results show that CC and HP interact dynamically, with the direction of causality depending on economic conditions and participants’ behaviors. On one hand, rising CC can stimulate housing demand, pushing HP upward. Conversely, when economic uncertainty rises, housing may serve as a hedge asset, attracting investment even amid weak CC, which further drives HP growth. Meanwhile, HP fluctuations also asymmetrically impact CC. Higher HP can boost household wealth and CC, but excessive HP growth may strain affordability, increasing mortgage burdens and reducing CC. These findings highlight the time-varying, reciprocal nature of CC-HP linkages, emphasizing the need for adaptive policy responses to stabilize both markets during economic turbulence.

## Introduction

The main aim of this paper is to investigate the dynamic time-varying interplay between consumer confidence (CC) and housing prices (HP) in China. Housing constitutes both a major household expenditure and a significant contributor to economic expansion [1]. Variations in HP exert substantial influence on macroeconomic stability [2] while simultaneously affecting consumption patterns and household financial conditions [3]. CC, which reflects households’ economic outlook and propensity to spend, serves as a fundamental determinant of housing demand and market behavior [4]. Therefore, the interaction between CC and HP is a core hub for understanding the fluctuations of the macroeconomic cycle and predicting financial stability, which has aroused a great deal of research interest [5]. Most studies suggest that CC influences HP through the expectations effect [6]. Improved CC raises optimism about incomes and the economy, boosting housing demand and pushing up HP, and vice versa [7,8]. However, an alternative view emphasizes the hedging effect of real estate [9]. From this perspective, even amid low CC, housing may continue to attract investment as a safe-haven asset, thereby sustaining or even elevating HP [10]. This behavior is motivated by the desire to hedge against uncertainty and secure long-term returns [11]. On the other hand, HP influences CC through the wealth effect and the debt effect. Rising HP enhances perceived net wealth and bolsters CC [12–15]. However, excessively high HP increases mortgage burdens, reduces affordability, and suppresses disposable income, thereby dampening CC [16–18]. Therefore, the relationship between these two variables is complex and contingent upon specific economic conditions and participant behaviors. Investigating the interaction mechanism between CC and HP holds significant theoretical and practical importance.

China’s housing market has experienced unprecedented growth over the past two decades, becoming the world’s largest [19] while demonstrating some of the most rapid price appreciation globally [20]. Between 2000 and 2024, prices in major cities increased approximately tenfold [21], driven by several unique characteristics of the Chinese market. First, the rapid advancement of urbanization and sustained high-speed economic growth have jointly created robust housing demand and strong purchasing power, serving as the core drivers behind China’s long-term HP appreciation. Second, information asymmetries create market conditions where individual buyers often make decisions based on psychological factors rather than rational analysis [5]. Third, limited alternative investment options in China’s developing capital markets have made residential property the preferred asset class for wealth preservation [22]. Fourth, deep-rooted cultural values associate homeownership with social status and marital stability, further intensifying housing demand [23]. These distinctive features make China’s housing market an ideal context for studying the relationship between consumer confidence and price dynamics.

This study contributes to the literature in the following manner. First, we systematically examine the bidirectional causality between consumer confidence and housing prices in China’s unique socioeconomic context, where housing serves multiple roles as consumption good, investment asset, and social status symbol. Our analysis reveals a complex feedback mechanism that differs substantially from patterns observed in mature markets. Second, by employing advanced time-varying Granger causality tests, we capture the non-linear dynamics of the CC-HP relationship across different economic cycles. Our innovative sub-sample analysis specifically identifies how major crises have differentially affected this relationship. Third, distinct from conventional unidirectional impact analyses, we systematically elucidate the complex bidirectional interactions between CC and HP. The identification of this dynamic interdependence provides a novel theoretical perspective for understanding the distinctive fluctuation patterns in emerging real estate markets.

The remainder of this paper proceeds with a review of relevant literature, followed by the presentation of the theoretical model underpinning the analysis. Subsequently, the research methodology is detailed, after which the data sources and descriptive statistics are introduced. The empirical findings and their implications are then discussed, culminating in a concluding section that summarizes key insights and policy recommendations.

## Literature review

### The impact of CC on HP

Existing research consistently demonstrates that CC exerts a substantial influence on HP. Armona et al. (2020) establish, through a US portfolio choice experiment, that HP expectations play a causal role in investment decisions [24]. Similarly, Loebs et al. (2015) illustrate how prevailing market sentiment shapes HP dynamics in the US [25]. Jin et al. (2014) further confirm that CC significantly drives HP fluctuations in the US market [26]. Biktimirov et al. (2024) highlight the role of news media sentiment in predicting HP changes [27]. Beyond the US, Peric et al. (2022) find that CC strongly affects short-term HP movements in European economies [28]. Yang et al. (2020) document a similar relationship in Seoul [29]. Cheong et al. (2020) show that contemporaneous sentiment predicts short-term housing returns in Malaysia [30]. Kholodilin et al. (2018) demonstrate CC’s predictive power for city-level HP in major German cities [31]. Kunze et al. (2020) confirm sentiment’s forecasting utility in the UK [32].

Most studies suggest that CC’s impact on HP is positive. Gholipour Fereidouni et al. (2017) find that CC reinforces the link between HP and consumption expenditure in the US [13]. Pyo (2022) show that sentiment shocks predict rising national-level HP in Korea [33]. Choi et al. (2021) observe a positive correlation between consumer sentiment and actual apartment transaction prices in South Korea [34]. However, some scholars challenge this consensus. Lowis et al. (2015) contend that macroeconomic fundamentals outweigh CC’s influence on HP [35]. André et al. (2021) suggest that exogenous CC shocks minimally affect US housing returns and volatility [36]. Kwakye et al. (2024) find CC’s role in South Africa’s real estate market marginal [37]. Bahmani-Oskooee et al. (2022) report short-term CC effects on HP in only 34 US states, with long-term persistence in just 13 [38]. Saydometov et al. (2020) note a negative relationship between future HP and negative sentiment, while positive sentiment shows no significant effect [39].

China’s rapid development has intensified scrutiny of the CC-HP nexus, yet findings remain mixed. Zhou (2018) observes that housing returns rise with contemporaneous sentiment but warns that high sentiment may precede lower future returns [40]. Hui and Wang (2014) document CC-driven HP appreciation in Hong Kong [41]. Zheng et al. (2016) emphasize investor expectations’ role in shaping HP dynamics [42]. Conversely, Hui et al. (2017) report a negative CC impact on Shanghai’s housing returns [43]. Shao et al. (2023) detect bidirectional causality, with sentiment negatively correlating with HP fluctuations over time [44].

### The impact of HP on CC

The impact of HP on CC is widely regarded by scholars as predominantly positive. Choi and Jo (2024) find that HP shocks have enduring effects on consumer prices through wealth and collateral channels across 41 US cities [12]. Case et al. (2005) conclude that the wealth effect from housing is substantial and that HP increases stimulate consumer expenditure, based on an examination of data from multiple countries [45]. Carroll et al. (2011) further establish that housing wealth affects consumption in both the short and long term [46]. Bonis and Silvestrini (2012) demonstrate a significantly positive impact of HP appreciation on consumption in OECD countries [47]. Cristini and Sevilla (2014) reaffirm that HP influence household consumption primarily through the wealth effect [48]. Gathergood (2012) indicates that consumption declines in response to falling HP for a significant proportion of households in the United Kingdom [49]. Qian (2023) shows that households that anticipate rising house prices report a greater readiness to increase spending in China [50]. Cevik and Naik (2025) confirms that household consumption responds positively and swiftly to changes in real HP in Europe [51]. Gholipour Fereidouni and Tajaddini (2017) find that CC has a positive effect on the association between housing wealth and consumption expenditure in the US [13]. Yin et al. (2021) show that an increase in HP can add to a household’s net worth and thus stimulate household consumption [14].

However, some studies hold the opposite view. Dong et al. (2021) find that rising HP in China inhibit financial leverage and subsequently weaken consumer expenditure [52]. Zhou et al. (2016) also observe a negative, albeit modest effect of housing wealth on consumption in China [53]. Yang et al. (2018) identify a negative correlation between HP and household consumption among homeowners with either one or two housing units in China [16]. Li and Zhang (2021) argue that the housing wealth-consumption relationship does not follow a simplistic pattern in China [18].

In addition, a group of studies presents more nuanced evidence, emphasizing conditional or bidirectional effects. Deng et al. (2022) find that the effect of HP increases is positive in the aggregate but redistributive, benefiting homeowners while negatively affecting renters in China [54]. Su et al. (2018) observe a significant long-term wealth effect in China that weakened after 2008, suggesting that excessively high HP began to constrain consumption in later years [55]. Apergis et al. (2014) identify a long-run bidirectional causal relationship between consumption and housing wealth in South Africa [56]. Sun et al. (2022) find that the impact of HP on consumption is asymmetric, with consumption moving in the opposite direction when HP rise but not when they fall [15].

In short, the previous relevant research is insightful, but there are still the following omissions for expansion. First, among the factors affecting HP, there are more studies on objective economic factors and fewer studies on subjective psychological factors, such as confidence, sentiment, expectation, etc. Second, there are few study on two-way causation of CC and HP, and most of results are positive. In different periods, the impact of CC on HP can be positive or negative. We utilize a sub-sample Granger causality test to examine the relationship between CC and HP over time. Combined with the different development backgrounds in different periods, specific issues are analyzed.

## Intertemporal capital asset pricing model

The relationship between CC and HP can be conceptualized through the lens of asset pricing theory. While the standard capital asset pricing model (CAPM) provides a static, single-period framework [57], the housing market is inherently intertemporal, with investment decisions based on expectations of future returns and risks. To capture this dynamic, we draw upon the intertemporal capital asset pricing model (ICAPM), originally developed by Merton [58], which argues that an asset’s expected return depends on its exposure to the market portfolio and state variables that describe the evolution of future investment opportunities. However, the unique structure of the Chinese housing market, characterized by significant speculation, information asymmetry, and a deep-rooted cultural preference for homeownership [5,22,23], necessitates a behavioral extension of the standard ICAPM. Following the work of by Cifarelli and Paladino on feedback trading in financial markets [59], we posit the existence of two distinct agent groups in the real estate market: (1) The rational group. This group consists of households with fundamental consumption demand. Their expectations about future housing prices are formed rationally based on available economic information. (2) The feedback group. This group comprises speculators and investors whose demand is driven primarily by past price trends rather than fundamental value. This behavior is prevalent in China’s market due to a lack of alternative investment channels and a widespread belief in the perpetual appreciation of real estate, fueled by historical price trends. CC is introduced as a key state variable in the ICAPM framework that influences the investment opportunity set and risk appetite of the rational group. It acts as a proxy for aggregate household optimism regarding future economic conditions, income growth and the future trajectory of HP. Thus, we model the rational group’s demand for housing as being positively influenced by CC:


$$R_{t}=\frac{E_{t-1}(HP_{t})-HP^{f}}{\mu (CC_{t})}$$ (1)

where *R*_*t*_ represents the proportion of real estate demanded by the rational group. $\mu \;\left(CC_{t}\right)$ is a positive and increasing function of perceived systemic risk, which is dampened by higher confidence. $E_{t-1}(HP_{t})$ denotes the conditional expectation of HP, while $HP^{f}$ signifies the risk-free return. If all consumers in the real estate market act rationally, *R*_*t*_ = 1, we would arrive at a standard pricing equation:


$$E_{t-1}(HP_{t})=HP^{f}+\mu (CC_{t})$$ (2)

This suggests that higher CC, by reducing risk perception and increasing demand, leads to higher expected and, consequently, realized prices. Conversely, the feedback group’s demand (*H*_*t*_) is modeled as a function of lagged prices:


$$H_{t}=\tau HP_{t-1}$$ (3)

where τ > 0, signifying that the feedback group increases demand following past price increases. The total market demand is the sum of both groups ($R_{t}+H_{t}=1$). Solving for the rational expectation by combining equations (1) and (3) yields:


$$E_{t-1}(HP_{t})=HP^{f}+\mu (CC_{t})-\tau \mu (CC_{t})HP_{t-1}$$ (4)

Equation (4) introduces the term $-\tau \mu (CC_{t})HP_{t-1}$, which captures the destabilizing potential of feedback trading. This theoretical framework provides a foundation for a time-varying relationship between CC and HP. The net effect depends on the relative strength of the rational demand channel and the feedback trading channel. When rational demand dominates, CC positively affects HP via the expectation effect. When speculative demand prevails, CC negatively influences HP through the hedging effect. This justifies the empirical use of rolling-window Granger causality tests to uncover how this relationship evolves over different periods, particularly during phases of market exuberance or stress dominated by one group or the other.

## Methodology

### Bootstrap full-sample technique

Although the traditional vector auto-regressive (VAR) approach can be used to investigate the correlation among time series, it is essential that these variables and the VAR process adhere to the standard normal distribution [60]. However, if this allocation cannot be fulfilled, it may undermine the reliability of the conventional VAR method [61]. To address this issue, Shukur and Mantalos (1997) introduced critical values derived from the residual-based bootstrap (*RB*) method, which can be applied in Granger causality examinations deviating from standard normal distributions [62]. Moreover, the RB method is conducive for analyzing datasets with limited sample sizes [63]. Additionally, Shukur and Mantalos (2000) proposed the likelihood ratio (*LR*) method, which can adjust power and size attributes [64]. Consequently, this study utilizes the revised-*LR* technique based on *RB* to explore causality among the time series. The VAR (*p*) system is represented by Equation (5).


$$Y_{t}=a_{0}+a_{1}Y_{t-1}+\ldots +a_{p}Y_{t-p}+\varepsilon_{t},t=1,2,3\ldots \ldots T$$ (5)

where *p* is selected via the Schwarz Information Criterion (SIC), facilitating the determination of the optimal lag order. Furthermore, *Y* could be alternatively denoted as $Y_{t}=(CC_{t},HP_{t}{)}^{\prime }$. Additionally, the association between CC and HP may be influenced by the money supply (M2) [65–67], which we consider as a control variable. Thus, Equation (5) can be reformulated as follows:


$$\left[\begin{array}{c} {}*20cCC_{t}\\ HP_{t} \end{array}\right]=\left[\begin{array}{c} {}*20ca_{10}\\ a_{20} \end{array}\right]+\left[\begin{array}{cc} {}*20ca_{11}(L) & a_{12}(L)\begin{array}{cc} {}*20c & a_{13}(L) \end{array}\\ a_{21}(l) & a_{22}(L)\begin{array}{cc} {}*20c & a_{23}(L) \end{array} \end{array}\right]\begin{array}{c} {}*20c \end{array}\left[\begin{array}{c} {}*20cCC_{t}\\ \begin{array}{c} HP_{t}\\ M2_{t} \end{array} \end{array}\right]+\left[\begin{array}{c} {}*20c\varepsilon_{1t}\\ \varepsilon_{2t} \end{array}\right]$$ (6)

Using the VAR (*p*) process described above, we can investigate the initial assumption that CC does not Granger-cause HP ($a_{21,k}=0,k\in [1,p]$). This assumption should be rejected if CC has noticeable effects on HP. Similarly, if HP significantly influences CC, the initial assumption that HP does not Granger-cause CC ($a_{12,k}=0,k\in [1,p]$) may be invalidated.

### Parameter stability test

The method described above assumes constant coefficients, a presumption that may not always hold in reality. When structural shifts in coefficients occur, relying on full-sample techniques may not produce reliable results. To ensure the accuracy of estimations, this study incorporates techniques Sup−F,Ave−F and Exp−F, as outlined by Andrews [68,69]. Technique Sup−F identifies structural changes within each sequence and the VAR (*p*) system, while the other two methods detect gradual coefficient changes over time. Additionally, the *L*_*c*_ statistics technique, developed by Nyblom (1989) [70] and Hansen (1992) [71], is used to evaluate the randomness of coefficient alterations. If abrupt structural changes arise in the variables and VAR (*p*) system, the causal relationship between CC and HP could fluctuate. Hence, relying solely on full-sample techniques proves inadequate, necessitating a more sophisticated sub-sample approach to capture this dynamic correlation.

### Bootstrap sub-sample technique

The sub-sample approach, introduced by Balcilar et al. (2010) [72], aims to discern the dynamic nature of the relationship between two variables. This method involves dividing the entire data set into smaller segments based on a specified rolling-window width, which are then continuously shifted from the beginning to the end. However, determining the optimal rolling-window width can be challenging, as using a smaller width may lead to inaccurate results, while a larger width could reduce the frequency of analysis. To address this issue, Pesaran et al. (2005) suggest that the rolling-window width should be set at a minimum of 20 when dealing with a VAR (*p*) system with changing coefficients [73]. The procedure in question can be succinctly summarized as follows:

First, given a total data length of *E* and a designated width of *f*, the endpoints of each segment will be f,f+1,…E. Second, each individual segment can be examined for its relationship using the *RB*-based revised-*LR* technique. Following this, the estimated results of this sub-sample technique can be obtained by sequentially calculating the *p*-values and *LR* statistics, as proposed by Balcilar et al.(2013). Additionally, the mean values of estimated outcomes ($N_{b}^{-1}\sum {}_{k=1}^{p}{\overset{\wedge }{a}}_{12,k}^{*}$ and $N_{b}^{-1}\sum {}_{k=1}^{p}{\overset{\wedge }{a}}_{21,k}^{*}$) indicate the influence of CC on HP and the impact of HP on CC. Moreover, in line with the work of Balcilar et al. (2010), this investigation carries out a 90% confidence interval with lower(5th quantiles of ${\overset{\wedge }{a}}_{12,k}^{*}$ and ${\overset{\wedge }{a}}_{21,k}^{*}$) and upper (95th quantiles of ${\overset{\wedge }{a}}_{12,k}^{*}$ and ${\overset{\wedge }{a}}_{21,k}^{*}$) bounds.

### Data source and descriptive analysis

This study examines monthly data from 2000M01-2025M03 to analyze the Granger causality relationship between CC and HP in China. China’s accession to the World Trade Organization in 2000 significantly accelerated its foreign trade growth and economic integration, which substantially enhanced CC [74]. Concurrently, rapid economic development, rising incomes, and large-scale urbanization have driven strong real estate demand, leading to sustained HP growth [75,76]. We measure CC using the China Consumer Confidence Index (CCI) from the National Bureau of Statistics of China, which ranges from 0 to 200, with 100 as the threshold distinguishing sufficient from insufficient confidence. Higher CCI values reflect stronger consumer expectations about the economy [77] and are associated with increased consumption, including housing purchases [78]. HP is represented by the national average housing price, also sourced from the National Bureau of Statistics of China. Expansionary monetary policy increases liquidity, driving up asset prices, including HP [65]. Additionally, money supply (M2) growth stimulates consumer credit expansion [66], which may alleviate liquidity constraints and boost CC [67], thereby influencing the CC-HP relationship. Given these dynamics, We include M2 as a control variable. All data are obtained from the National Bureau of Statistics of China.

**Fig 1:**
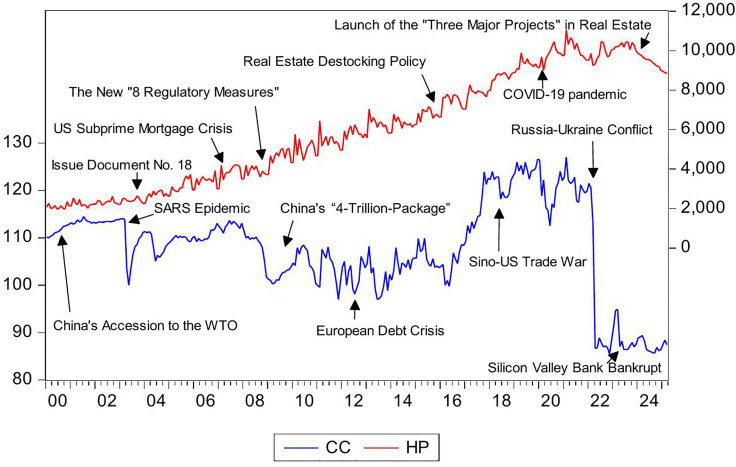
The trends in CC and HP.

Fig 1 presents the trends in CC and HP in China since 2000. The real estate market has served as a key economic driver to promote growth and stimulate consumption [79]. This role was formalized in 2003 when the State Council designated housing as a pillar industry through Document No. 18. From then until 2022, the housing market experienced rapid expansion with steadily rising HP. Despite government measures to cool the market, HP maintained an upward trend except for temporary declines during the 2008 financial crisis, 2011 European debt crisis, and 2020 COVID-19 pandemic. In contrast, CC showed significant fluctuations. After China joined the WTO in 2001, CC initially rose but dropped sharply during the 2003 SARS outbreak. The 2008 financial crisis caused another dramatic decline. From late 2008 to mid-2016, CC fluctuated around 100 before rising to around 120 due to economic improvements in productivity, technology, and incomes [80]. This level persisted for over four years until COVID-19 caused a sharp drop. A partial recovery followed, but the Russia-Ukraine conflict in April 2022 triggered a steep decline from 120.5 to 86.7. By 2025, CC remained below 90 amid economic slowdown. The comparison reveals that CC and HP often moved in opposite directions. This countercyclical pattern suggests their relationship is more complex than traditional models capture. Therefore, a sub-sample analytical approach is necessary to properly examine the Granger causality between these variables.

Table 1 presents the key descriptive statistics for our three focal variables. The mean values stand at 108.98 for CC, 5784.279 for HP, and 1046353 for M2, with each series exhibiting significant volatility as demonstrated by their respective ranges between minimum and maximum values. The distributional analysis reveals important characteristics. CC displays negative skewness of −0.399, indicating a left-tailed distribution, while both HP and M2 show positive skewness of 0.248 and 0.620 respectively. Regarding kurtosis, CC demonstrates leptokurtic properties with a value exceeding 3, whereas HP and M2 exhibit platykurtic distributions with values below 3. The Jarque-Bera test results confirm the non-normality of all three variables at standard significance thresholds. Therefore, using the traditional Granger causality test may not be appropriate.

**Table 1:** Descriptive statistics.

	Mean	Median	Maximum	Minimum	Standard Deviation	Skewness	Kurtosis	Jarque-Bera
CC	108.98	109.8	127	85.5	8.853	−0.399	3.392	9.437^***^
HP	5784.279	5586.272	11029.54	1984.396	2743.198	0.248	1.74	21.843^***^
M2	1046353	838542.4	3135322.30	121220.4	815149.5	0.620	2.162	26.712^***^

Notes: ^***^ denotes significance at the 1% level.

## Empirical results

We conducted unit root tests [81,82] to assess the stability of CC and HP. The results indicate that both variables become stable after first differencing. Subsequently, employing the VAR model with lag length determined by the Schwarz information criterion, we examined the causal relationships between CC and HP. The optimal lag length for both variables is found to be 2. The results in Table 2 show that CC does not exert a causal effect on HP, and vice versa. The full-sample approach assumes no structural changes in the time series, implying that the causal links between variables remain constant throughout the entire time span. Nevertheless, the presence of structural breaks in the series renders the causality between CC and HP unreliable and non-constant. The parameter stability test, employing the Sup−F,Ave−F, Exp−F, and *L*_*c*_ tests [68,69], was conducted to assess the stability of the data. Results from Table 3 indicate that for CC, the null hypothesis of parameter constancy is rejected at a 1% significance level of of Sup−F and Exp−F, and at a 5% significance level for Ave−F tests. Similarly, for HP, the null hypothesis is rejected at a 1% significance level under the Sup−F,Ave−F and Exp−F tests. This suggests that the time series of CC and HP exhibit non-constant behavior. Additionally, significant *L*_*c*_ statistics further confirm the lack of stability in the VAR model’s time series data.

**Table 2:** Full-sample Granger causality tests.

Tests	H0: CC does not Granger cause HP		H0: HP does not Granger cause CC	
	Statistics	*p*-values	Statistics	*p*-values
Bootstrap *LR* test	0.6855	0.6787	3.9892	0.1246

Notes: *p*-values are calculated from 10,000 bootstrap repetitions.

**Table 3:** The parameter stability test.

Tests	CC		HP		VAR system	
	Statistics	*p*-value	Statistics	*p*-value	Statistics	*p*-value
*Sup-F*	22.076^***^	0.001	45.706^***^	0.000	26.368^***^	0.000
*Ave-F*	6.763^**^	0.030	12.769^***^	0.000	11.798^***^	0.000
*Exp-F*	7.225^***^	0.004	17.946^***^	0.000	9.682^***^	0.000
*Lc*					0.953^***^	0.000

Notes: *p*-values are calculated from 10,000 bootstrap repetitions.

*** represent significance at 1% levels, ** represent significance at 5% levels.

Given the constraints of a full-sample causality test for this data set, a more advanced approach is warranted, such as the sub-sample rolling causality test. This method offers an advantage by employing a 24-month width, ensuring robustness [64]. To ensure the reliability of the causation, we also examine the data using widths of 20, 28, and 32 months [73]. The results are similar to those observed at the 24-month widths, indicating consistent outcomes. By introducing this novel method, the study elucidates the connection between CC and HP by scrutinizing the null hypothesis. Furthermore, it enables the determination of the directional effects between the two variables.

**Fig 2:**
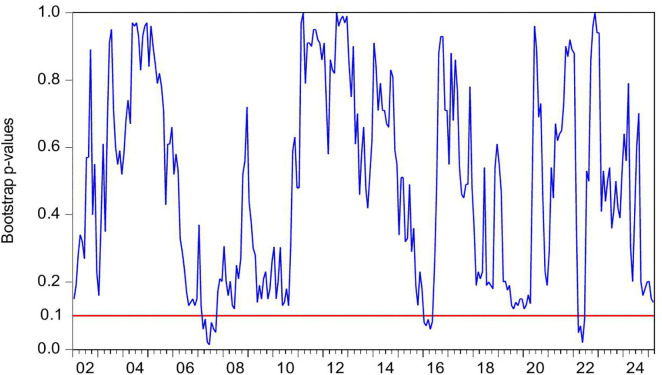
The *p*-values from the rolling-window estimation test whether CC does not Granger cause HP.

**Fig 3:**
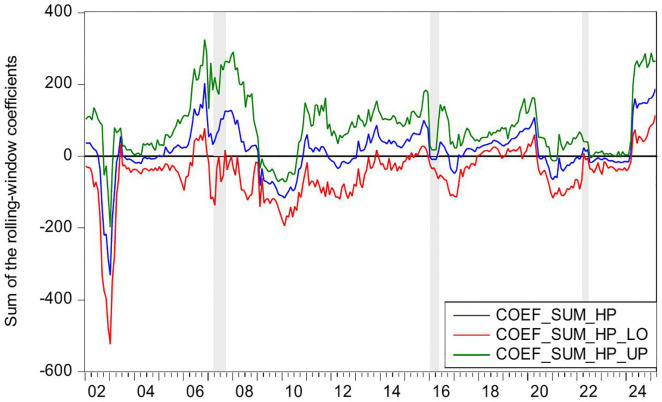
The coefficients for the effect of CC on HP.

Fig 2 presents the statistical significance of CC as a Granger-cause of HP, as evidenced by the reported *p*-values. The null hypothesis (no causal relationship) is rejected at the 10% significance level (p < 0.1), confirming the existence of Granger-causality between these variables. Fig 3 further illustrates the directional impact of this causal relationship. When the blue trend line rises above the zero threshold, it indicates a positive influence of CC on HP. Conversely, values below zero suggest a negative directional impact. Combining these figures, we observe significant positively causality from CC to HP during 2007M03-2007M09 and 2022M03-2022M06, CC’s impact on HP is negative in 2016M01-2016M05.

During the period of 2007M03-2007M09, China witnessed a concurrent rise in CC and HP, supported by strong macroeconomic fundamentals. The economy expanded at 11.4% in 2007, marking its highest growth rate since 1992. CPI climbed to 4.8% while M2 grew by 16.7%. The country recorded a historic trade surplus of 262.2 billion dollars, the largest globally, alongside foreign direct investment inflows totaling 74.7 billion dollars. China’s foreign exchange reserves increased from 1.202 trillion dollars in March to 1.43 trillion dollars by September 2007, becoming the first nation to hold reserves exceeding one trillion dollars. The renminbi appreciated 6.5% against the US dollar, attracting substantial speculative capital. The domestic stock market experienced an unprecedented rally, with the Shanghai Composite Index rising from 998 points in June 2005 to a peak of 6124 points by October 2007. Driven by these multiple factors, China experienced a significant surge in CC. Meanwhile, HP also rose sharply. Nationwide HP surged 14.8% year on year. Leading cities saw particularly sharp increases, with Shenzhen up 45.2%, Beijing 44.6%, and Guangzhou 36.7%. During this period, the positive impact of CC on HP can be explained from the following three perspectives. First, rising CC reflects strong expectation and confidence in economic conditions and income growth, stimulating demand for durable goods such as housing and thereby driving up HP [6–8]. Second, China’s per capita GDP exceeded 2000 US dollar in 2007, leading to a rapid expansion of the middle class. This demographic shift triggered a surge in demand for upgraded housing among middle- and high-income households [83]. Third, The 2007 stock market boom generated substantial profits for retail investors, some of whom redirected their gains into real estate investments [84]. In summary, China’s soaring HP were fueled by strong CC, rising incomes and wealth, and the growing middle class’s demand for improved housing conditions.

During the period of 2016M01-2016M05, China’s CC declines while HP rise. During the first half of 2016, China’s CCI experiences a phased drop, falling from 104 in January to 99.8 in May. From June 2015 to January 2016, the Shanghai Composite Index plunges by 43%, significantly eroding the wealth of households. In January 2016, another stock market circuit breaker triggers a four-day loss of 6 trillion yuan in market value, further damaging investor confidence. Additionally, GDP growth slows to 6.7% in the first half of 2016, and the manufacturing Purchasing Managers’ Index remains below the expansion-contraction threshold for multiple consecutive months. Capacity reduction in traditional industries such as steel and coal leads to layoffs, pushing the urban surveyed unemployment rate up to 5.2%. The macroeconomic slowdown results in weaker income growth and reduced CC. However, HP surge sharply. From late 2015 to early 2016, the State Council introduces multiple policies to reduce housing inventory and stabilize the market. Between January and May 2016, average new HP in China’s first-tier cities rise by 20%−30% year-on-year, with Shenzhen recording the highest increase at 47%. In key second-tier cities, Hefei sees a 39% price jump in six months, while Nanjing experiences a 21% rise. In cities like Hangzhou, new housing projects sell out immediately upon launch, forcing buyers to enter lotteries to purchase homes. Additionally, developers aggressively acquire land, resulting in 110 record-high land auctions nationwide in the first half of 2016. During this period, the negative impact of CC on HP can be explained as the following. China’s economic growth and household income expansion decelerate, while profit margins in the real economy decline, leading to weakened CC and increasingly pessimistic expectations [85]. However, real estate in first-tier cities maintains an investment return rate exceeding 8%, significantly higher than the sub-5% returns in the real economy [86]. Under loose monetary conditions with M2 growth sustaining at 13%, traditional safe-haven assets such as government bonds no longer outpace inflation [87]. As a result, real estate emerges as a natural monetary shelter, fulfilling capital’s demand for hedging [9]. Surveys in Wenzhou reveal a 200% year-on-year surge in property purchases by manufacturing business owners. In Beijing, the proportion of all-cash transactions in the secondary housing market climbs sharply to 41% in the first half of 2016. In essence, this period reflects a unique economic landscape where abundant liquidity coexists with declining real economy returns, driving capital toward real estate as a hedge [10,11].

During the periods of 2022M03-2022M06, China’s CC and HP declines. The Russia-Ukraine conflict erupted on 24 February 2022, has triggered a surge in global energy and food prices, supply chain disruptions, and market volatility. The sharp rise in oil prices drives up transportation and living costs, eroding household purchasing power. Disruptions to Ukraine’s grain exports exacerbate food inflation, straining the budgets of low- and middle-income consumers. Meanwhile, supply chain disruptions further dampen spending on big-ticket items. Heightened geopolitical uncertainty also leads to a sharp decline in CC. In April 2022, the CCI drops sharply to 86.7, reflecting a 26.5 point decline from the previous month. This represents the most significant monthly decrease since March 2020 when the COVID-19 pandemic began. China’s real estate market also experiences a downturn, characterized by widespread HP declines and a sharp contraction in transaction volumes. In the first half of 2022, the HP nationwide drops by 8.6% year-on-year. During the same period, both the sales area and sales value of commercial properties register significant declines, falling by 22.2% and 28.9% year-on-year, respectively. During this period, declining CC exerts downward pressure on HP, demonstrating a positive impact of CC on HP. This relationship can be explained as follows. First, the Russia-Ukraine conflict has heightened global uncertainty risks [88]. Concurrently, China’s GDP growth has slowed, pandemic impacts have weakened employment, and household income expectations have declined, leading to reduced home-purchasing capacity and CC [89]. Consequently, investors and home buyers adopt a wait-and-see approach, delaying or canceling purchase plans, thereby suppressing HP [90]. Second, contrary to the optimistic expectations for the real estate market in 2016, China’s HP have lost fundamental support by 2022 due to slowing urbanization, peaking demographic dividends, and high inventory in some cities, shattering the myth of perpetually rising HP [91,92]. Additionally, defaults by major developers like Evergrande and widespread unfinished projects have severely eroded market trust [93]. Topics such as mortgage defaults and surging foreclosed properties on social media further dampen CC. These factors accelerate the spread of pessimism, prompting consumers not only to postpone home purchases but also to prepay mortgages, making deleveraging a new trend and accelerating HP declines [94].

**Fig 4:**
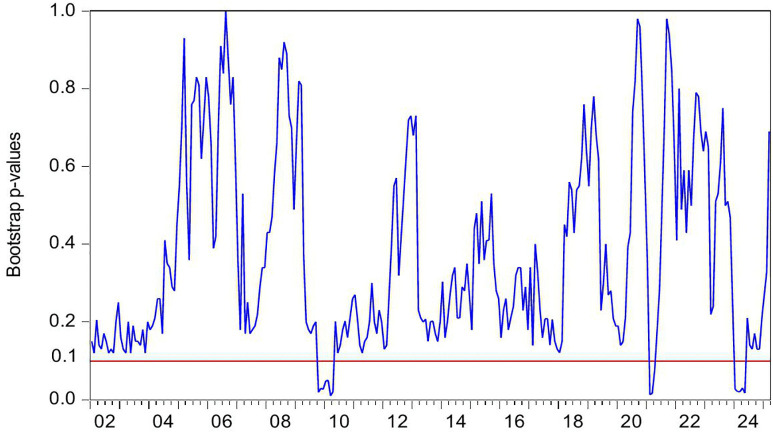
The *p*-values from the rolling-window estimation test whether HP does not Granger cause CC.

**Fig 5:**
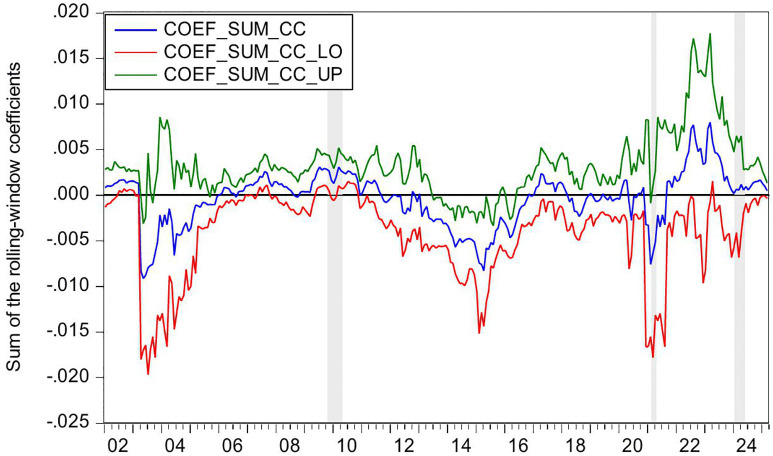
The coefficients for the effect of HP on CC.

Fig 4 demonstrates the statistically significant Granger-causal relationship from HP to CC, with the null hypothesis of non-causality being rejected at the 10% significance level (p < 0.1). Fig 5 further illustrates the directional impact of this causal relationship. Where positive values (blue line > 0) indicate HP’s stimulative effect on CC, while negative values (blue line < 0) reflect inhibitory impacts, with the zero threshold serving as the critical demarcation for directional significance. By integrating these two figures, we observe that HP exerts a positive effect on CC during the periods of 2009M10-2010M04 and 2024M01-2024M05. Conversely, HP demonstrates a negative impact on CC during the periods of 2021M02-2021M04.

During the periods of 2009M10-2010M04, both HP and CC rose. In the second half of 2009, driven by China’s “4-Trillion-Package”, China’s economy not only began a strong recovery but also saw its real estate market rebound remarkably from the shadow of the global financial crisis. The stimulus package positively impacted the property sector in three key ways: First, credit conditions loosened significantly, with new RMB loans reaching a record 9.59 trillion yuan in 2009, of which developer loans and mortgage lending accounted for a substantial share. Commercial banks relaxed lending criteria for real estate firms, while homebuyers enjoyed preferential mortgage rates at 30% discounts, directly fueling housing demand [95]. Second, infrastructure investments generated spillover effects [96]. Large-scale projects such as high-speed rail and metro systems drove up HP along their routes [97]. Third, excess liquidity spurred asset allocation demand. With stock markets volatile, substantial capital flowed into real estate as a store of value [98]. These factors collectively drove rapid HP increases in 2009–2010. In 2009, national commercial housing sales reached 937.13 million square meters, up 42.1% year-on-year, while sales revenue surged 75.5% to 4.3995 trillion yuan. HP rose by 24%, a historical high, with quarter fourth accounting for 62% of the annual increase. Land sales revenue hit 1.59 trillion yuan, up 63% year-on-year. The upward trend continued into the first half of 2010. Meanwhile, CC rebounded alongside improving economic indicators. China’s CCI rose from 103 in quarter fourth 2009–107 in quarter first 2010, marking a post-crisis peak. During this period, the positive impact of HP on CC can be explained through the wealth effect [12,13]. The specific transmission channels are as follows: First, the rise of HP directly increases the property realization income of property owners. In addition, higher property valuations enable banks to offer larger home equity loans, more favorable refinancing terms, and better credit conditions. This improved liquidity significantly strengthens households’ purchasing power for both durable goods and discretionary spending, thereby elevating CC [14]. Second, some households incorporate potential future property appreciation into long-term consumption plans, enhancing CC via anticipated income gains [15]. Third, rising HP and media coverage of a booming real estate market reinforce public expectations of economic improvement, further elevating CC [5,27].

During the periods of 2021M02-2021M04, HP rise, but CC falls. The first months of 2021 mark a critical transition in China’s post-pandemic economic recovery toward structural adjustment. Loose credit conditions and heightened inflation expectations contribute to a short-term HP surge. Nationwide HP increase by 5% year-on-year, while commercial housing sales rise by 48.1%. From January to April, the average land premium rate in 100 major cities climbs to 18.7%, reinforcing HP growth expectations. However, CC weakens during this period. The CCI declines from 127 in February to 121 in April, reflecting growing economic uncertainty despite the housing market boom. In this period, the HP has a negative effect on CC. The rise of HP leads to the deterioration of household balance sheet, and eventually leads to the decline of CC and consumption contraction, forming a negative transmission chain of HP-debt-CC [16]. First, rapid HP appreciation expands mortgage debt and drives up household sector leverage. China’s household debt to GDP ratio peaks at 62.3% in 2021. Elevated debt service burdens reduce disposable income thereby depressing CC [17]. Second, higher HP raise down payment requirements and monthly mortgage payments. This forces households to cut discretionary spending and weakens CC [18]. Third, rising HP expectations strengthen precautionary saving motives. The household savings rate rebounds to 34.6% in 2021. Meanwhile, excessive housing asset concentration reaches 80% of total household assets in 2021. This creates a liquidity mismatch where asset appreciation coexists with tightened cash flow further suppressing CC [79].

During the period of 2024M01-2024M05, both HP and CC falls. In the first half of 2024, China’s HP declines by 5.7% year-on-year, marking the sharpest drop in nearly a decade. housing sales volume down 19.0%, while total sales value down 25.0%. National real estate development investment falls 10.1%, and residential land supply and demand in 300 major cities shrink by over 30% year-on-year. This widespread HP correction results from multiple interacting factors. Economic growth slows to a record low of 5.2%, directly weakening market confidence. Urbanization deceleration combined with population aging reduces the size of core homebuying demographics. Anticipation of impending property tax implementation heightens market anxiety, as rising holding costs accelerate investor exits. These mutually reinforcing factors collectively drive the HP downturn in the first half of 2024. In addition, China’s CC, showing a continued downward trend, the index fell from 89 to a new low of 86. During this period, the decline in HP led to a decline in CC. The positive impact of HP on CC can be explained as follows. First, Chinese household wealth remains heavily concentrated in real estate. Falling HP lead to significant asset depreciation. The evaporation of middle-class wealth directly weakens consumption capacity [99]. Additionally, declining collateral values reduce households’ ability to obtain consumer credit through property mortgages. Collectively, these factors amplify the negative wealth effect and suppress CC [100]. Second, HP declines reinforce pessimistic expectations about economic prospects. Households grow concerned about potential income reductions or rising unemployment risks, dampening CC [101].

### The robustness test

This study conducts a robustness check by replacing M2 with interest rates as the primary control variable. Lower interest rates reduce borrowing costs, thereby stimulating housing demand and elevating HP, whereas rate increases exert a contractionary effect on the market.

**Fig 6:**
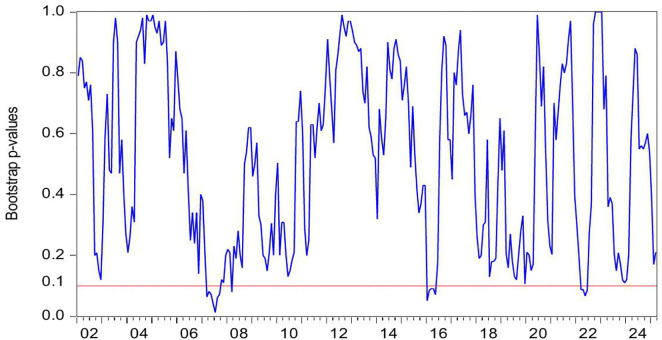
The *p*-values from the rolling-window estimation test whether CC does not Granger cause HP in the robustness test.

**Fig 7:**
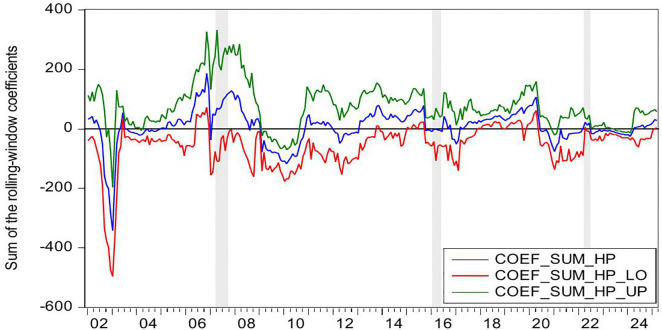
The coefficients for the effect of CC on HP in the robustness test.

**Fig 8:**
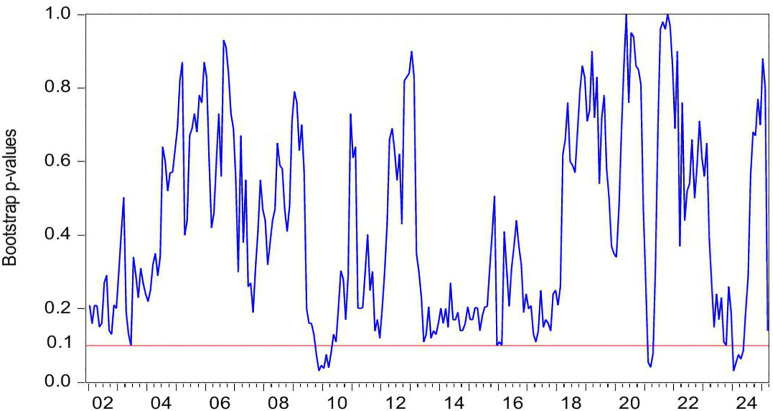
The *p*-values from the rolling-window estimation test whether HP does not Granger cause CC in the robustness test.

**Fig 9:**
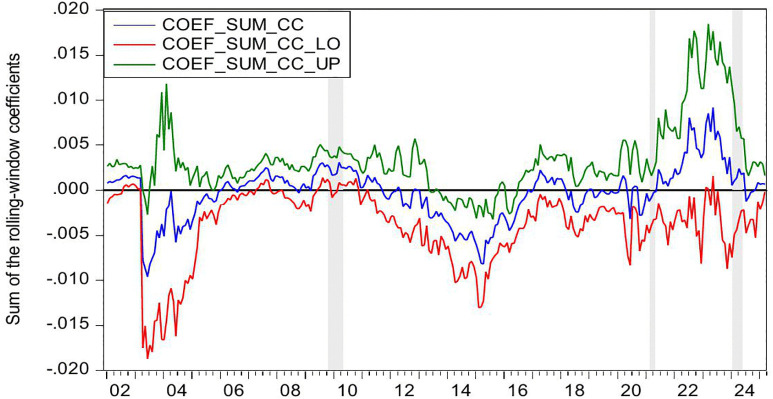
The coefficients for the effect of HP on CC in the robustness test.

As illustrated in Figs 6–9, the regression outcomes remain consistent with the prior findings when altering the core control variables. The robustness test confirms our core findings hold under different control variables, validating the methodology’s reliability.

## Comparative analysis with India

India serves as an ideal comparative case for China due to their shared characteristics in economic development, real estate market dynamics, population size, and integration into globalization. This parallel makes India a scientifically justified reference point for examining similar trends in emerging economies. This study investigates the bidirectional relationship between the Consumer Confidence Index (CCI) and the Real House Price Index for India (RHPII) using monthly data from 2013M08 to 2025M06. The CCI, sourced from the Wind Database, measures fluctuations in Indian household CC, while the RHPII, obtained from the Organisation for Economic Co-operation and Development (OECD), tracks India HP.

**Fig 10:**
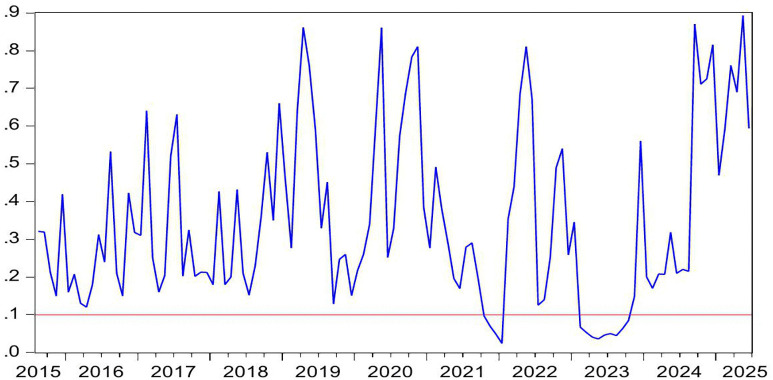
The *p*-values from the rolling-window estimation test whether CC does not Granger cause HP in India.

**Fig 11:**
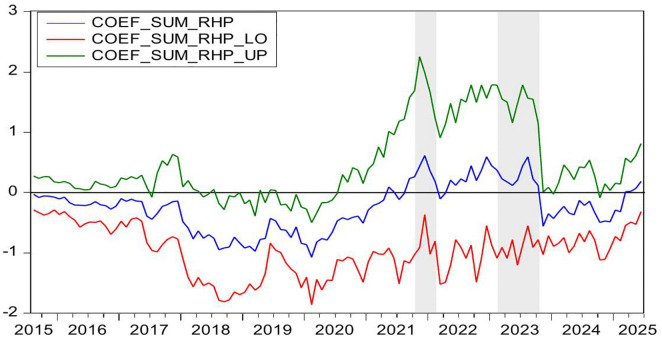
The coefficients for the effect of CC on HP in India.

**Fig 12:**
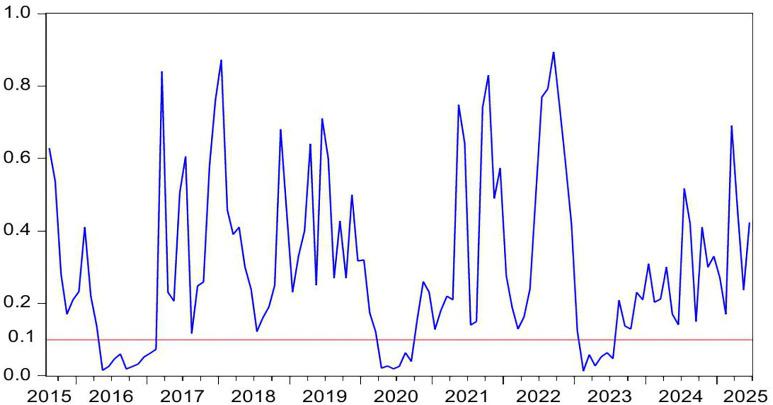
The *p*-values from the rolling-window estimation test whether HP does not Granger cause CC in India.

**Fig 13:**
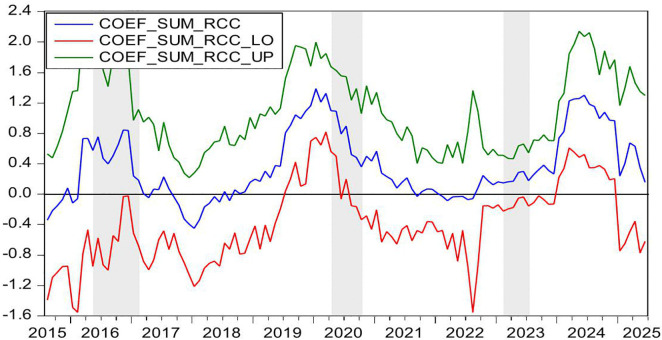
The coefficients for the effect of HP on CC in India.

Figs 10–13 present the empirical results of the bidirectional interaction between CC and HP in India. The analysis reveals two key findings during the study period. First, the study reveals robust two-way interactions between CC and HP in India. CC significantly influences HP movements, while HP changes concurrently feedback into CC dynamics. Second, the Indian CC and HP exhibit a self-reinforcing feedback loop. Rising HP boosts CC through the wealth effects, while heightened CC in turn stimulates greater housing demand by the expectations effects, further driving up HP. These results are inconsistent with findings from China. In China, there are not only wealth effects and expectation effects, but also hedging effects and debt effects.

Possible explanatory factors for this cross-country variation may include: First, the differential maturity of mortgage markets explains why high HP create debt effects in China but not in India. China’s developed mortgage system facilitates widespread home purchases through bank lending, locking households into long-term debt burdens. Conversely, India’s underdeveloped formal credit channels constrain leverage, inadvertently preventing debt entrapment despite high HP. Second, unlike China’s financialized housing market that acts as a key investment asset, India’s real estate remains constrained by structural barriers. High transaction costs, legal uncertainties, and cultural preferences for gold limit housing’s appeal as a speculative vehicle. Indian property stays anchored to consumption needs rather than becoming a driver of capital appreciation. This divergence explains why India lacks China’s the hedging effects despite similar CC pressures.

## Conclusions and policy implications

This study employs rolling window analysis to reveal the complex time-varying relationship between CC and HP, which operates through 4 primary transmission channels. First, expectation effect channel. Strengthened CC fosters greater optimism among households about the future, enhancing their homebuying willingness and credit access. This, in turn, stimulates housing demand and subsequent increases in HP. Second, hedging effect channel. During periods of low CC, if real estate yields surpass those of alternative investments, real estate emerges as a preferred hedge asset. Consequently, capital flows from the real economy into the property market, leading to rising HP. Third, wealth effect channel. Rising HP increases household wealth, which in turn boosts CC. Fourth, debt effect channel. When HP growth consistently outpaces income growth, the attendant rise in household debt eventually curbs CC by elevating mortgage burdens, while also dampening CC. The impact of CC on HP is positive when the expectation effect dominates the hedging effect, and negative in the converse scenario. Similarly, HP exerts a positive influence on CC when the wealth effect prevails over the debt effect, with the relationship turning negative otherwise.

Based on the impact of CC on HP, the following three suggestions are proposed. First, a decentralized platform should be established to automatically collect and verify real transaction data, generating an unbiased price index. When abnormal market fluctuations are detected, the system should temporarily suppress overpriced listings and impose a cooling off period to stabilize market conditions. Second, a digital advisory tool should be developed to compute and display key financial metrics, such as price-to-income ratios, mortgage payment burdens, and historical price trends, thereby reducing irrational decision-making influenced by market sentiment. Third, introduce futures contracts and price insurance mechanisms to allow developers to hedge against construction cost volatility and homebuyers to mitigate asset depreciation risks. This dual approach enhances market stability by aligning risk management tools with both supply-side and demand-side needs, fostering a more resilient and balanced housing market.

To prevent high HP from exacerbating household debt burdens and dampening CC, institutional arrangements should be implemented across three key dimensions. First, establish income adaptive mortgage loan mechanisms, including differentiated debt-to-income caps, income linked mortgages, and smart repayment adjustment systems, to mitigate household debt risks at the source. Second, introduce phased property rights allocation, enabling homebuyers to acquire ownership incrementally, thereby smoothing housing costs over time and reducing repayment pressure. Third, strengthen relief mechanisms such as personal bankruptcy systems and emergency loan relief funds to provide structured risk resolution for debt-distressed households.

## Supporting information

S1 Dataset Data of CC, HP and M2. (XLSX)

S1 Appendix Sensitivity analysis with alternative rolling-window lengths. (DOCX)
