# Supplementary material for: Dynamic interdependence between consumer confidence and housing prices: Evidence from bootstrap rolling window causality tests
Source: PLoS One. 2026 Jan 12;21(1):e0340354. doi: 10.1371/journal.pone.0340354 (PMC12795388; doi:10.1371/journal.pone.0340354)
Supplement: S1 Appendix — (DOCX) [file pone.0340354.s002.docx]

**Fig 14-Fig 17: Results of the bootstrap sub-sample rolling-window causality test for 20 month windows.**

**Fig 14**

**

**

**Fig 15**

**

**

**Fig 16**

**

**

**Fig17**

**

**

**Fig18-Fig 21: Results of the bootstrap sub-sample rolling-window causality test for 28 month windows.**

**Fig 18**

**

**

**Fig19**

**

**

**Fig 20**

**

**

**Fig 21**

**

**

**Fig 22-Fig 25: Results of the bootstrap sub-sample rolling-window causality test for 32 month windows.**

**Fig 22**





**Fig 23**





**Fig 24**





**Fig 25**
